# Special Issue on Ruthenium Complexes

**DOI:** 10.3390/molecules22020255

**Published:** 2017-02-08

**Authors:** Ileana Dragutan, Valerian Dragutan, Albert Demonceau

**Affiliations:** 1 Institute of Organic Chemistry “C.D. Nenitescu”, Romanian Academy, 202B Spl. Independentei, 060023 Bucharest, P.O. Box 35-108, Romania; 2 Department of Chemistry, University of Liège, Sart-Tilman (B.6a), 4000 Liège, Belgium

The organic chemistry of ruthenium has been one of the most vigorously growing research areas over the past decades. Considerable effort has been extended towards the design and application of a broad series of ruthenium complexes, which culminated with the development by Ryoji Noyori (2001 Nobel Prize for Chemistry) of chiral ruthenium catalysts for stereoselective hydrogenation reactions [1], and the discovery by Robert H. Grubbs (2005 Nobel Prize for Chemistry) of well-defined ruthenium–benzylidene catalysts for olefin metathesis [2].

The aim of this special issue was to provide an overview of recent trends in ruthenium complex chemistry, hereby underlining its growing importance in the development of anticancer drugs and applications in catalysis, polymers, materials science, and nanotechnology [3].

The submitted contributions can be roughly grouped into four categories: (1) synthesis of new ruthenium complexes, (2) applications in organic synthesis and catalysis, (3) photocatalysis and dye-sensitized solar cells, and (4) medicinal chemistry. With the exception of the article of Igor T. Chizhevsky et al. [4], which deals with the synthesis and characterization of new *closo*-ruthena-carborane complexes with a dioxygen ligand, most of the contributions actually fall into two or even three categories, so that the following summary of the special issue is by no means a strict classification.

As one of these categories, organic synthesis and homogeneous catalysis have constituted for a long time a major field of application of ruthenium complexes. Along this line, Vincenzo Piccialli [5] reviews in this issue the chemistry of ruthenium tetroxide and perruthenate with a special emphasis on oxidation of alcohols and hydrocarbons, dihydroxylation of alkenes, oxidative cleavage of C–C double and triple bonds. New processes, synthetic applications, theoretical studies and unusual transformations mediated by these species are also covered in this review. The asymmetric transfer hydrogenation of imines catalyzed by the Noyori–Ikariya half-sandwich ruthenium complexes has been surveyed by Petr Kačer et al. [6]. Aspects highlighted in this review include the role of the *N*-arylsulfonyl moiety and that of the *η*^6^-coordinated arene of the catalytic systems, and also the effect of structural modifications on the imine substrate. The supremacy of ruthenium–benzylidene complexes in olefin metathesis is illustrated by a contribution of Hermanus C. M. Vosloo et al. [7], who synthesized new chelating pyridinyl-alcoholato ligands and incorporated them into the second-generation Grubbs catalyst. The influence of the ligand substituents on the thermal stability, activity, selectivity and lifetime of these complexes in the metathesis of 1-octene has been investigated. On the other hand, the development of recyclable catalysts is nowadays a major trend towards sustainable chemistry. In their excellent micro-review, Dong Wang and Didier Astruc [8] focused their attention on the fabrication of recoverable magnetic nanoparticle-supported ruthenium complexes and their catalytic applications in various organic syntheses.

With many potential applications in photochemistry, ruthenium complexes have always been in the focus of synthetic organometallic chemists. A contribution to this category was made by Ludovic Troian-Gauthier and Cécile Moucheron [9] reviewing the photophysics reported in the literature for mononuclear, binuclear and polynuclear ruthenium complexes bearing ligands with extended aromaticity. Of these, binuclear complexes with extended π-systems finding practical applications in photocatalysis are treated in more detail. In the same direction, Adewale O. Adeloye and Peter A. Ajibade [10] have surveyed recent advances in the chemistry of ruthenium polypyridine complexes that have been designed and synthesized for use as photosensitizers in dye-sensitized solar cells. Special attention was paid to the correlation of the ligand structure with the photophysical, electroredox and power conversion efficiency of representative ruthenium polypyridyl complexes as well as to ruthenium complexes containing new polypyridine ligands with long-range electron transfer motifs such as alkenyl, alkynyl and polyaromatic donor functionalities.

The fourth category comprises possible applications of bioactive ruthenium complexes. Lusiane Maria Bendhack et al. [11] have reviewed the effects of ruthenium-derived NO donor complexes on the control of vasodilatation and arterial pressure. In particular, the crucial importance of the chemical structure of these ruthenium complexes for their vascular effects has been emphasized. Fernando Rogério Pavan et al. [12] reported on the in vitro anti-tuberculosis activity and cytotoxicity of ruthenium compounds encapsulated in nanostructured lipid systems composed of cholesterol, surfactant, and aqueous phase. Finally, Claudio L. Donnici [13] investigated the in vitro antifungal activity of a series of ruthenium dithiocarbamate complexes against different fungal species of clinical interest and related to invasive fungal infections. Very promising results were obtained and a preliminary structure–activity relationship was established.

At this point, we would like to thank all contributors for the submission of their articles, which allow this special issue to provide a comprehensive view of the current developments in the chemistry of ruthenium complexes, thus proving that modern chemistry is today indeed more ruthenium-dependent than ever before. Our sincere acknowledgements also go to the numerous referees who spent their time and attention to give helpful advice for improvements. Thanks are also due to the editorial staff of *Molecules* for their valuable support in perfecting this Special Issue.
